# FTH promotes the proliferation and renders the HCC cells specifically resist to ferroptosis by maintaining iron homeostasis

**DOI:** 10.1186/s12935-021-02420-x

**Published:** 2021-12-29

**Authors:** Wanye Hu, Chaoting Zhou, Qiangan Jing, Yancun Li, Jing Yang, Chen Yang, Luyang Wang, Jiayu Hu, Huanjuan Li, Hairui Wang, Chen Yuan, Yi Zhou, Xueying Ren, Xiangmin Tong, Jing Du, Ying Wang

**Affiliations:** 1 Laboratory Medicine Center, Department of Laboratory Medicine, Zhejiang Provincial People’s Hospital, Affiliated People’s Hospital, Hangzhou Medical College, Hangzhou, 310014 Zhejiang China; 2 GCP Clinical Research Center, Affiliated Hangzhou First People’s Hospital, Zhejiang University School of Medicine, Hangzhou, 310006 Zhejiang China; 3 Graduate School, Bengbu Medical College, Bengbu, 233030 Anhui China; 4 School of Pharmacy, Zhejiang University of Technology, Hangzhou, 310014 Zhejiang China; 5 Department of Central Laboratory, Affiliated Hangzhou First People’s Hospital, Zhejiang University School of Medicine, Hangzhou, 310006 Zhejiang China

**Keywords:** FTH, Hepatocellular carcinoma, Ferroptosis, Iron homeostasis, Mitochondria

## Abstract

**Background:**

Ferroptosis is a newly identified type of programmed cell death, which preferentially targets iron-rich cancer cells such as hepatocellular carcinoma (HCC). Ferritin heavy chain (FTH) is a major iron storing nanocage to store redox-inactive iron, and harbors ferroxidase activity to prevent the iron-mediated production of ROS. Our previous studies have demonstrated that FTH acts as a protective role to increase the cellular resistance to ferroptosis. However, the specific role of FTH in the development of HCC and ferroptosis resistance remains unclear.

**Methods:**

The indicated databases were used for bioinformatics analysis. The abilities of cell proliferation, migration were measured by cell proliferation assay, transwell assay and wound healing assay. The levels of reactive oxygen species (ROS), lipid peroxide, free iron, mitochondrial superoxide, mitochondrial morphology and mitochondrial membrane potential (MMP) were determined by DCF-DA, C11-BODIPY, mitoSOX, mitoTracker, JC-10 and TMRM staining, respectively. The mitochondrial oxygen consumption rate was monitored by the Seahorse XF24 Analyzer.

**Results:**

The pan-cancer analysis was performed and showed that FTH expression is upregulated in multiple cancers, such as LIHC, CHOL, HNSC, compared to corresponding normal tissues. In addition, the level of serum ferritin is positively associated with the progression of hepatitis, cirrhosis liver and hepatocellular carcinoma. Further investigation shed light on the strong correlation between FTH expression and tumor grades, cancer stages and prognosis of HCC. Importantly, the proteins interaction network elucidated that FTH is involved in iron homeostasis maintenance and lysosomal-dependent degradation. Enforced expression of FTH accelerates proliferation, migration and endows HCC cells specifically resistant to ferroptosis, but does not protect against cell death caused by cytotoxic compounds like oxaliplatin, irinotecan, and adriamycin. Mechanically, FTH reconstituted cells exhibit diminished peroxides accumulation, reduce mitochondrial ROS level, attenuate the impaired mitochondrial respiratory and rescue the mitochondrial homeostasis. Notably, FTH expression boosts tumorigenic potential in vivo with increased PCNA staining and lesser lipid peroxides generation.

**Conclusion:**

These results provide new insights that FTH acts as an oncogene in the carcinogenesis and progression of HCC, and is hopeful to be a potential target for therapeutic intervention through ferroptosis.

**Supplementary Information:**

The online version contains supplementary material available at 10.1186/s12935-021-02420-x.

## Introduction

Liver cancer, one of the most common neoplastic malignancies, is characterized by high incidence, postsurgical recurrence and poor prognosis [1, 2]. Hepatocellular carcinoma (HCC) accounts for 70–90% of primary liver cancer, caused a heavy burden of disease on people and is estimated to be the third leading cause of cancer-related death worldwide [2]. The main risk factors for HCC include chronic hepatitis, alcohol addiction, metabolic liver disease and so on [3]. Early monitoring can partly prevent these risk factors to increase the chance of a potential cure. However, HCC surveillance and prevention are substantially underutilized even in countries with adequate medical resources [3, 4]. At present, the main clinical treatments of early-stage HCC are local ablation, surgical resection or liver transplantation [5]. Nevertheless, only a small cohort of patients with HCC are diagnosed at an early stage, most are typically diagnosed at advanced stages when the tumors are unresectable, contributing to the failure of the surgical treatment. The pathogenesis of HCC is very complex and HCC is insensitive to traditional radiotherapy or chemotherapy [6]. Currently, available systemic treatments for HCC remain limited in terms of overall survival [7]. Therefore, there is an urgent need to further study the pathogenesis of HCC and facilitate the identification of novel diagnostic targets and treatment strategies.

Ferroptosis is a newly identified type of programmed cell death, which is morphologically, biochemically, and genetically distinct from other forms of cell death. Ferroptosis is typically characterized by the abnormal accumulation of lipid hydroperoxides resulting from the excess overload of ferrous iron, which can be specifically rescued by iron chelators [8, 9]. Since the discovery of ferroptosis, both the molecular mechanisms of ferroptosis and its potential application have become popular research topics. Several proteins such as SLC7A11/xCT, glutathione peroxidase 4 (GPX4), nuclear factor erythroid 2-related factor 2 (NRF2), ACSL4, LPCAT3 enzymes, and some small molecules such as disulfiram, sorafenib, iFSP, zalcitabine and withaferin, have been identified implicating in the regulation of ferroptotic cell death [10–13]. As previously mentioned, amplifying cancer cells exhibit more dependence on iron than the non-malignant cells, which is termed iron addiction [14], revealing an appealing therapeutic approach for triggering ferroptosis in iron-rich tumors. Although the primary pathway of ferroptosis has been established, the specific molecular mechanism underlying this regulatory network remains largely unknown. Therefore, identifying the precise regulatory mechanisms in ferroptosis is important, which may provide insights into new therapeutic avenues in cancers.

Ferritinophagy is a newly identified recycling process for the autophagic turnover of ferritin in lysosomes, which is mediated by autophagic cargo receptor nuclear receptor coactivator 4 (NCOA4), and leads to the initiation of ferroptosis [15]. Ferritin is comprised of 24 subunits of light chain (FTL) and heavy chain (FTH) isoforms and plays a central role in iron metabolism by chelating up to 4,500 iron atoms per spherical heteropolymer. Meanwhile, FTH harbors ferroxidase activity which involves the conversion of Fe^2+^ to Fe^3+^ for storing excess cellular iron in ferritin nanocages. Thus, FTH is believed to prevent the iron-mediated production of reactive oxygen species (ROS) and tissue damage. Fang et al. reported a remarkable observation that high iron diet in FTH knockout mice caused cardiac injury and hypertrophic cardiomyopathy through induction of ferroptosis, demonstrating that ferritin protects against cardiac ferroptosis and subsequent heart failure [16]. Mumbauer et al*.* found that knockdown of FTH in the larval wing discs leads to drastic growth defects, ROS accumulation in wing discs, severe mitochondrial defects and ferroptosis [17]. Although increasing evidence indicated that FTH involves in the regulation of ferroptosis, the role of FTH in the progression of hepatocellular carcinoma and the resistance of ferroptosis remain obscure.

In the current study, we investigated the expression and the role of FTH in HCC through bioinformatics analysis. In addition, we have discovered that the level of serum ferritin is positively associated with the progression of hepatitis, cirrhosis liver and hepatocellular carcinoma. Furthermore, we constructed the FTH overexpression cells and explored the impacts of FTH on the biological phenotype of HCC cells. We provided evidence that FTH facilitates proliferation in HCC cells, eliminates mitochondrial oxidative stress and favors resistance to ferroptosis of HCC cells both in vitro and in vivo for the first time. Of note, we revealed the role of FTH in molecular biological characteristics of HCC, and presented a potential target towards antitumor activity through ferroptosis.

## Materials and methods

### Oncomine

Oncomine database (http://www.oncomine.org) is a publicly accessible online cancer microarray database for DNA or RNA sequences analysis. In our study, we used the Oncomine database to show transcriptional expressions of FTH between different cancer tissues and their corresponding adjacent normal control samples under the parameters: *P* value < 0.01, fold change ≥ 1.5, gene rank top 10%, data type mRNA. The difference of transcriptional expression of FTH was compared by Student’s *t*-test.

### Timer

The Tumor Immune Estimation Resource (TIMER) (https://cistrome.shinyapps.io/timer/) contains six tumor-infiltrating immune subsets in 10,897 tumors from 32 cancer types. We explored the correlation of FTH1 with infiltrating immune cells (CD8^+^ T cells, CD4^+^ T cells, B cells, dendritic cells macrophages, and neutrophils) and tumor purity in HCC patients.

### Univariate logistic regression analysis

We used univariate Cox regression analysis to determine the association between HCC patients’ overall survival (OS) and FTH1 expression. FTH1 expression was statistically significant in Cox regression analysis when *P* value < 0.05.

### Expression and survival analysis

The association of FTH expression and overall survival in different cancers was conducted by The Gene Expression Profiling Interactive Analysis (GEPIA) (http://gepia.cancer-pku.cn/index.html). We also compared the expressions of FTH in LIHC based on sample types, tumor grades and cancer stages by UALCAN (http://ualcan.path.uab.edu/index.html). Statically significant difference was considered under the condition of *P* value < 0.05.

### Patients and treatment

Retrospective study was conducted on outpatients and inpatients in Zhejiang Provincial People’s Hospital by searching the medical record system. 1026 healthy persons from physical examination, and 283 hepatitis, 283 cirrhosis, 593 hepatocellular carcinoma patients hospitalized in the People's Hospital of Zhejiang Province from January 2012 to November 2020 were enrolled. Their serum ferritin levels were measured. All the diagnostic criteria were based on the World Health Organization (WHO) criteria. This study was approved by the Ethics Committee of People's Hospital of Zhejiang Province, and written informed consent was exempted because of the retrospective design of this study. Differences were considered statistically significant at *P* < 0.05 by Student’s *t*-test. The statistical analyses were performed with the Statistical Package for the analysis of variance (ANOVA) using GraphPad Prism version 5.0.

### Enrichment analysis of FTH1 related genes

20 genes related to FTH1 were obtained by using the GENE MANIA website (https://genemania.org/) and then inputted into the Metascape (https://metascape.org/) to get the available enrichment analysis results.

### Cell lines

HCCLM3 and MHCC97H human hepatocellular carcinoma cells were preserved and passaged by our laboratory and cultured in DMEM medium (Hyclone, Logan, UT, USA) supplemented with 10% fetal bovine serum (Gibco, Grand Island, USA), 1% penicillin (100U/mL) and streptomycin (100 μg/mL) (Beyotime, Shanghai, China). All cells were cultured in a humidified incubator at 37 ℃, 5% CO_2_.

### Reagents and antibodies

(1S,3R)-RSL3 (RSL3), Dihydroxyacetone (DHA), Oxaliplatin, Irinotecan, Adriamycin were purchased from Selleck Chemicals (Houston, TX). The antibodies to Ferritin Heavy Chain (FTH) (ab75973), Ferritin Light Chain (FTL) (ab109373), Transferrin Receptor (TFR) (ab80194), Heme Oxygenase 1 (HO1) (ab68477), β-Actin (ab8226) and proliferating cell nuclear antigen (PCNA) (ab118197) were obtained from Abcam (Cambridge, MA). RIPA buffer, Cell Counting Kit-8 (CCK-8) kit and DAPI were obtained from Beyotime (Shanghai, China). 2’,7’-dichlorofluore scindiacetate (DCF-DA) was acquired from Sigma-Aldrich (St. Louis, USA). Rhodamine B-[(1, 10-phenanthroline-5-yl)-aminocarbonyl] benzyl ester (RPA) was attained from Squarix Biotechnology (Germany). FerroOrange was purchased from Dojindo Laboratories (Japan). The bicinchoninic acid protein assay kit, Lipid Peroxidation Fluorescent Probe (C11 BODIPY 581/591), mitoTracker Red and Tetramethylrhodamine methyl ester (TMRM) fluorescence probes were obtained from Thermo Fisher Scientific (Waltham, MA). JC-10 was acquired from Solarbio Life Sciences (China).

### Lentiviral packaging and transfection

Full-length FTH cDNA was ordered from Sino Biological (Beijing, China), amplified by ApexHF HS DNA Polymerase (Accurate Biotechnology, Hunan), subcloned into pLVX-IRES-Neo lentivirus vector by Seamless Cloning kit and verified by sequencing. The recombinant lentiviral plasmids were co-transfected with pMD2.G, pSPAX2 into 293 T cells to produce recombinant lentiviral. For transfection, HCCLM3 and MHCC97H cells were plated in a 24-well plate, and co-incubated with the corresponding lentivirus for 8 h. Then the stable cell lines were screened by G418 (1000 μg/mL) for 7 days.

### Cell proliferation assay

Cells were seeded in a 96-well plate at a density of 0.2 × 10^4^ cells/well and cultured in the medium containing 10% FBS. 10 μL CCK-8 solution (MultiSciences, Hangzhou) was added respectively into each well at 0, 24, 48, 72 h, and incubated for 1.5 h at 37 ℃ in the incubator. The optical density was detected at 450 nm using a microplate reader. GraphPad Prism 5.0 software was utilized to analyze the data and draw the line chart of cell proliferation ability.

### Dynole-monitoring of cell growth

To determine the change of cell growth in real-time, 0.2 × 10^4^ HCCLM3 and MHCC97H cells were seeded into Cytoview Z-Plate (CytoView-ZTM 96-Black, USA) in DMEM medium supplemented with 10% FBS, and maintained in 5% CO_2_ incubator. Then the plate was monitored continuously for 96 h via the MaestroZ real-time cell analyzer (Axion BioSystems, USA). The electrical impedance is positively correlated with the number of cells and collected every 15 min in each cabinet.

### Transwell assay

HCCLM3 and MHCC97H cells with or without FTH overexpression were collected and seeded in the upper chamber. 500 µL media supplemented with 5% FBS was added to the bottom chamber. After incubation for 36 h, the cells were fixed with 4% paraformaldehyde for 15 min, and stained with 0.1% crystal violet for 20 min. Then, cells on the upper chamber surface were gently wiped with cotton swabs, and the cells across the chamber were photographed by the Nikon DS-Ri2 microscope (Japan). The experiments were performed in triplicates, and five fields per chamber were photographed.

### Wound healing assay

Cells were seeded in a 6-well plate (NEST Biotechnology) and cultivated until 90% cell confluence. The scratch wounds were generated by scraping the cell monolayers with a sterile 10 µL tip. Detached cells were removed by washing with PBS 3 times. Then, cells were cultured in DMEM with 5% FBS. Images were acquired with Nikon DS-Ri2 microscope (Japan) at 0, 24 and 48 h.

### Cell viability assay

The cell viabilities of HCC cells were detected by CCK-8 as the manufacturer’s instructions. Briefly, 2 × 10^4^ cells per well were plated in three replicates in a 96-well plate, and incubated with various concentrations of RSL3 (0–2 µM), DHA (0–45 µM), cystine-free medium, Oxaliplatin (0–50 µM), Irinotecan (0–80 µM), Adriamycin (0–72 µM) separately. For the rescue assay, cystine-free medium and RSL3 were co-treated with various pharmacological inhibitors including ferrostatin-1 (fer-1, 1 µM), DFO (100 µM), GSH (0.5 mM), N-acety-L-cysteine (NAC, 1 mM), Z-VAD-FMK (5 µM). After the treatments, 10 µL CCK-8 was added to each well, and incubated for 1.5 h. The absorbance of 450 nm in each well was detected by a microplate reader. The respective curves of drug toxicity to HCC cells were graphed by GraphPad Prism 5.0.

### Determination of ROS levels

Cells in a 6-well plate (5 × 10^5^ cells/well) were treated with various concentrations of RSL3 (0–2 µM), DHA (0–45 µM) and cystine-free medium for 8 h. Cells were then stained with DCF-DA (4 µM) or C11-BODIPY (5 µM) for 30 min in dark at 37 ℃. Finally, the fluorescence intensity was detected.

### Western blot (WB) assay

The HCC cells with indicated treatment were collected and lysed in RIPA buffer for 10 min on ice. Cell lysates were centrifuged at 12,000 rpm at 4 ℃ for 10 min. The protein concentration was determined by the bicinchoninic acid protein assay kit. Protein samples were heated in SDS-loading buffer at 95 ℃ for 10 min, and separated by 10% SDS-PAGE. Then, proteins were transferred onto PVDF membranes (Bio-Rad, Hercules, CA) and blocked in 5% skim milk for 1 h. The membranes were incubated with corresponding antibodies at 4 ℃ overnight. Subsequently, the protein bands were washed 3 times and incubated with indicated secondary antibodies (TransGen Biotech) for 1 h at room temperature (RT). ECL chemiluminescence system was utilized to capture protein density (Bio-Rad, Hercules, CA).

### Confocal microscopy assays

2 × 10^5^ cells were seeded in four-well chambers dish and incubated with cystine-free medium, then cells were stained with RPA (2 µM), FerroOrange (1 µM), DCF-DA (4 µM), mitoSOX Red (5 µM), mitoTracker Red (200 nM), and JC-10 (3 µM), which were used for detecting the free iron, cellular ROS, mitochondrial superoxide, mitochondrial morphology and mitochondrial membrane potential, respectively. After staining in the incubator for 30 min, cells were washed with PBS 3 times, and representative images were photographed by the Leica confocal microscope.

### Oxygen consumption rate (OCR) determination

Cells (2 × 10^4^ cells/well) were seeded in XFe24 seahorse cell culture microplate (Seahorse Bioscience) for 24 h, and the XFe24 sensor cartridges were hydrated overnight. The cells were treated with cystine-free medium for 8 h, then the cell medium was replaced by basic seahorse DMEM supplemented with glucose (10 mM), sodium pyruvate (1 mM) and glutamine (2 mM). Subsequently, the cell culture microplate was then kept in the CO_2_-free incubator for 1 h, and OCR was measured in real-time with the sequential injection of oligomycin (1.5 μM), carbonyl cyanide-chlorophenylhydrazone (CCCP) (2.0 μM) and Antimycin A/Retenone (0.5 μM) by the Seahorse XFe 24 Bioanalyzer (Seahorse Bioscience).

### Animal experiments

Six-week-old male BALB/c nude mice were purchased from the Shanghai SLAC Laboratory Animal Co, Ltd. Mice were randomly allocated into two groups, then 2 × 10^6^ control and FTH ectopic expression cells were subcutaneously injected into the right dorsal flank, respectively. The tumor volume and body weight were recorded every 2 days. Mice were euthanized by CO_2_ inhalation (with a flow rate 20% per min) and tumor tissues were collected for subsequent experiments. All animal procedures in this study were approved by the Animal Ethical Committee of Zhejiang provincial people’s hospital.

### Immunohistochemistry assay and H&E staining

Tumor tissues were fixed in 4% paraformaldehyde (Leagene Biotechnology, China) overnight, embedded in paraffin, and then sliced into 4 μm segments. After deparaffinized and dehydrated, the slides were subjected to antigen retrieval, blocked and stained with anti-PCNA antibody (1:100), anti-FTH antibody (1:100), anti-c-Caspase-3 antibody (1:100) overnight at 4 ℃. After incubation with secondary antibody, the slides were visualized by the DAB staining kit. The H&E staining was carried out according to the manufacture’s instructions (Leagene Biotechnology, China). All the tissue slides were photographed by the Nikon DS-Ri2 microscope (Japan).

### Statistical analysis

The data were shown as the mean ± S.D. Differences between the two groups were assessed with Student’s *t*-test and comparisons among multi groups were evaluated by the analysis of variance (ANOVA) using GraphPad Prism version 5.0. The value of *P* < 0.05 was considered statistically significant.

## Results

### FTH expression is upregulated in multiple cancers

**Fig. 1:**
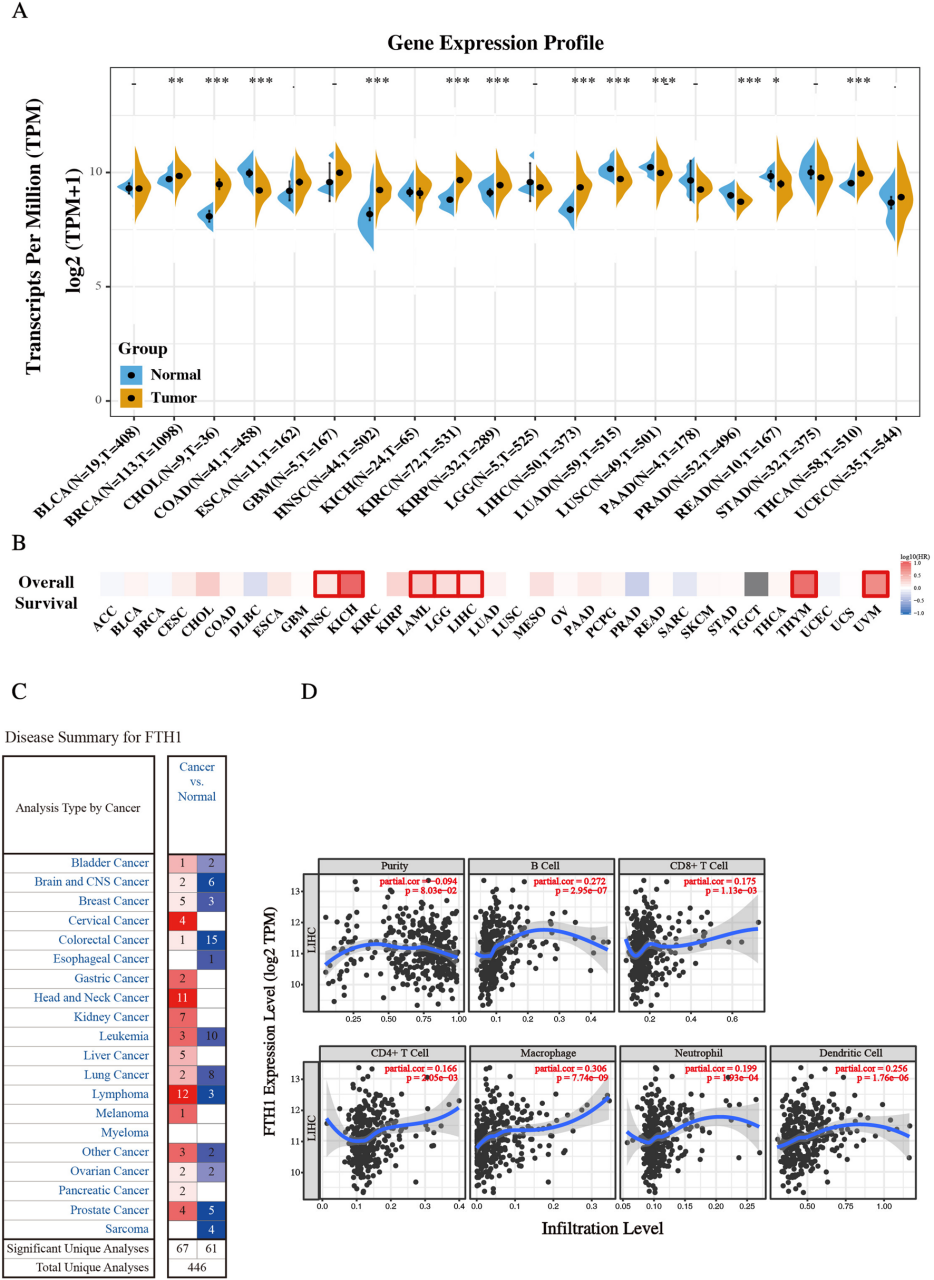
The expression level of FTH1 in various tumors. **A** The expression of FTH1 in various cancer types was summarized by the SangerBox tool. **B** Heatmap showed the relationship of overall survival and FTH1 in varieties of cancers (GEPIA database). **C** Transcriptional expression profiles of FTH1 in multiple cancer types (Oncomine database). **D** Cox regression analysis of FTH1 expression with immune infiltration level in LIHC. Scatter plots were used to perform the immune infiltration level in different immune cells. Purity Spearman's rank correlation test was utilized to obtain *P* values and partial correlation (cor) values. (Values are represented as mean ± SD. ^★^*P* < 0.05, ^★★^*P* < 0.01, ^★★★^*P* < 0.001 versus indicated groups)

To identify the molecular biological characteristics of FTH in cancers, the expression of FTH was investigated in tumors and its adjacent normal samples using the Sanger BOX online website based on the samples from The Cancer Genome Atlas (TCGA) and GTEx projects. As shown in Fig. 1A, FTH was significantly upregulated in most tumors, including Cholangiocarcinoma (CHOL), Neck squamous cell carcinoma (HNSC), Kidney renal clear cell carcinoma (KIRC), Kidney renal papillary cell carcinoma (KIRP), Liver hepatocellular carcinoma (LIHC), Thyroid carcinoma (THCA) and so on, compared with respectively adjacent normal tissues. That indicated FTH might play a critical role in the process of tumorigenesis in these types of cancers. We additionally employed the prognostic value of FTH in cancers within the RNA sequencing data in TCGA to assess how FTH expression relates to prognosis in a range of cancer types. The gene expression profile data revealed that FTH expression represents a high hazard ratio (HR) in the OS of multiple cancer types, especially in LIHC, Brain lower grade glioma (LGG), Acute myeloid leukemia (LAML), Kidney chromophobe (KICH), Head and neck squamous cell carcinoma (HNSC), Thymoma (THYM) and uveal melanoma (UVM) (Fig. 1B). These results demonstrated that FTH expression might act as a potential indicator of tumor prognosis. The expression of FTH was also evaluated by the Oncomine database (Fig. 1C). Consistent with the data from TCGA, FTH mRNA expression was excessively upregulated in hepatocellular carcinoma compared to that of normal liver tissues in multiple datasets. In Roessler Liver and Liver 2 datasets, FTH mRNA was upregulated with 1.984-fold (*P* = 3.14E−45) and 1.948-fold (*P* = 1.92E−05) increase in HCC samples [18]. The result from the Mas Liver dataset showed that there was 1.718-fold (*P* = 5.15E−06) increase of FTH mRNA expression in HCC tissues [19]. Other results from Wurmbach and Chen Liver also observed the increase of FTH mRNA in HCC samples (Table 1) [20, 21]. Additionally, we analyzed the correlation of FTH1 expression with infiltrating immune cells and tumor purity. The result displayed in Fig. 1D revealed that the expression of FTH has significantly positive correlation with B cells (r = 0.272, *P* = 2.95e−07), CD8 + T cells (r = 0.175, *P* = 1.13e−03), CD4 + T cells (r = 0.166, *P* = 2.05e−03), macrophages (r = 0.306, *P* = 7.7e−09), neutrophils (r = 0.199, *P* = 1.93e−04), and dendritic cells (r = 0.256, *P* = 1.76e−06) in HCC. Collectively, these results together showed that FTH is significantly up-regulated in primary HCC tissues compared to normal samples.

**Table 1:** Significant changes of FTH1 expression in transcription level between HCC and normal liver tissues (From ONCOMINE database)

	Types of HCC VS. Liver	Fold Change	*P* value	t-test	Refs.
FTH1					
	Hepatocellular Carcinoma	1.984	3.14E-45	16.235	Roessler Liver 2 [18]
	Hepatocellular Carcinoma	1.718	5.15E-06	4.992	Mas Liver [19]
	Hepatocellular Carcinoma	1.948	1.92E-05	4.756	Roessler Liver [18]
	Hepatocellular Carcinoma	2.086	1.62E-04	4.403	Wurmbach Liver [20]
	Hepatocellular Carcinoma	1.176	0.001	3.103	Chen Liver [21]

*HCC* Hepatocellular carcinoma, *FTH1* Ferritin heavy chain 1

### High FTH expression is linked to high risk and poor prognosis in LIHC

**Fig. 2:**
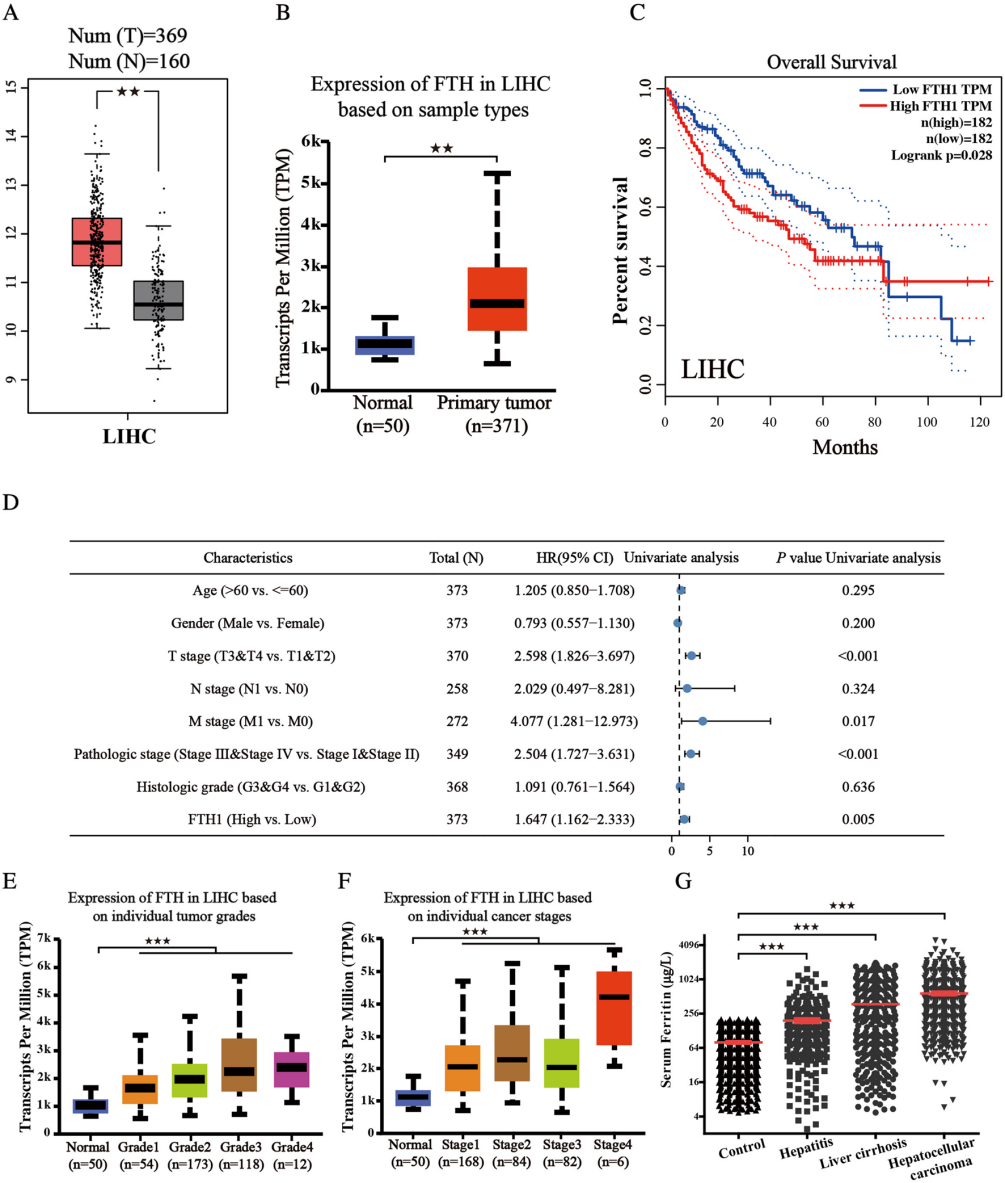
High FTH expression is linked to high risk and poor prognosis in HCC. **A**, **B** mRNA expressions of FTH1 were discovered to be overexpressed in primary hepatocellular carcinoma tissues compared to adjacent normal tissues using GEPIA and UALCAN databases. **C** Higher mRNA expression of FTH1 was associated with shorter OS in hepatocellular carcinoma patients (GEPIA database). **D** The overall survival in a cohort of 593 hepatocellular carcinoma patients was analyzed by univariate analysis. **E**, **F** The association of FTH1 expression with cancer stages and tumor grades (TCGA samples). **G** The level of serum ferritin in the indicated groups was determined. (Values are represented as mean ± SD. ^★^*P* < 0.05, ^★★^*P* < 0.01, ^★★★^*P* < 0.001 versus indicated groups)

To better distinguish the prognostic and potential therapeutic value of FTH in LIHC patients, we characterize the mRNA expression of FTH in LIHC from the TCGA database and GEPIA website. As shown in Fig. 2A, B, FTH is highly expressed in LIHC compared with the normal group. The prognosis of OS in LIHC was analyzed according to the expression of FTH from the GEPIA, which indicates that high expression of FTH in LIHC is associated with poor prognosis (Fig. 2C). A univariate Cox model revealed that clinical T, M, pathologic stage and high FTH expression had a positive correlation with HCC patients’ OS (Fig. 2D). We also verified the relationship between mRNA expression of FTH with patients’ cancer stages and tumor grades by UALCAN whose resources were based on level 3 RNA-seq and clinical data from 31 cancer types of TCGA database. The results from subgroup analysis showed that the expression of FTH was significantly higher in individual tumor grades and cancer stages compared with normal tissues, and patients who were in more advanced cancer stages tended to with higher mRNA expression of FTH. The highest mRNA expressions of FTH were found in Grade 4 and Stage 4 (Fig. 2E, F). After clinicopathological parameters were found to be significantly associated with the expression of FTH, we analyzed the level of serum ferritin in the following four different groups of people: the normal healthy people, the patients with hepatitis, the patients with liver cirrhosis and the patients with LIHC. The data indicated a strong positive and close relationship between the level of FTH and the progression of hepatocellular carcinoma (Fig. 2G). Collectively, these results revealed that FTH expression is linked to high risk and poor prognosis in LIHC and might act as an oncogene in carcinogenesis and progression.

### FTH promotes the proliferation and migration of HCC cells

**Fig. 3:**
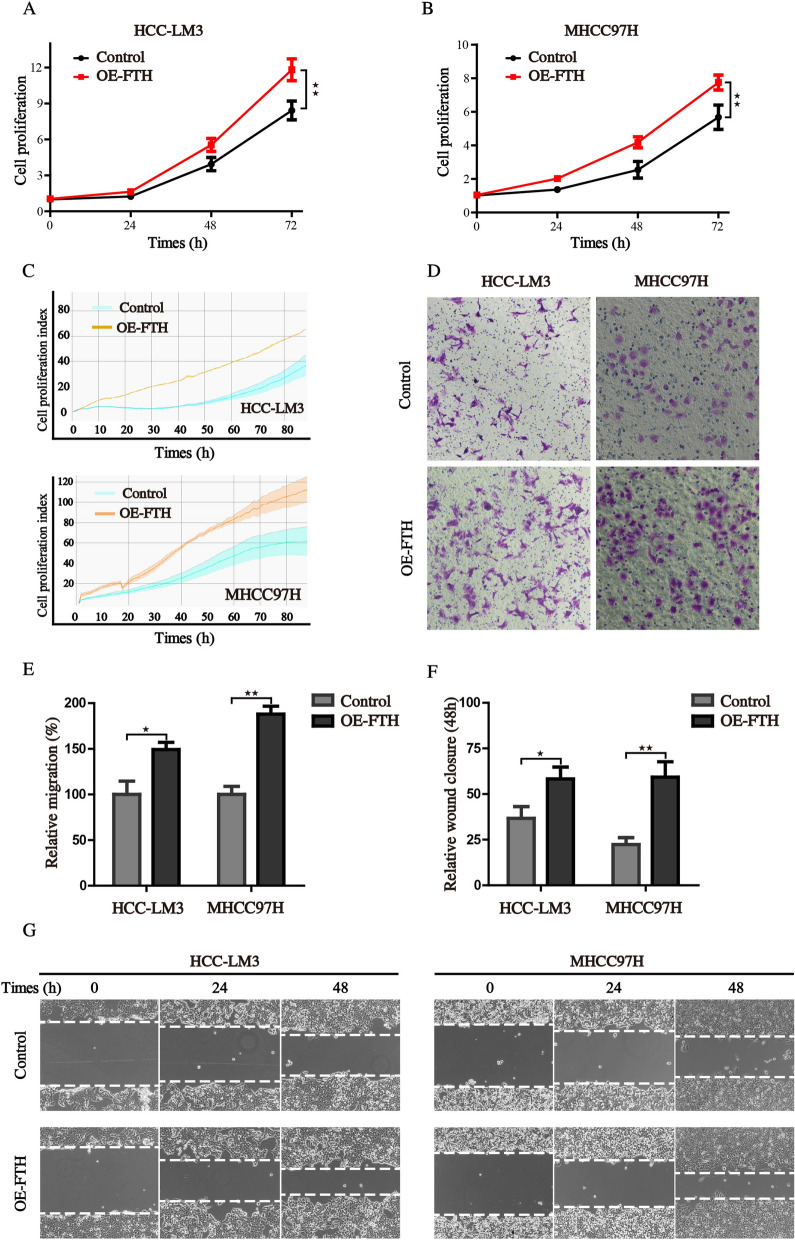
FTH promotes the proliferation and migration of HCCcells. **A**, **B** Cell proliferation ability of control and FTH overexpression was assessed using the CCK8 assay. **C** Real-time cell proliferation ability was monitored by the MaestroZ Real-Time Cell Analyzer. **D** The invasive capability of cells was evaluated by transwell assay. **E** The statistical histogram of transwell assay. **F**, **G** Wound healing assay was employed to detect the migration capacity of control and FTH overexpression cells. (Values are represented as mean ± SD. ^★^*P* < 0.05, ^★★^*P* < 0.01 versus indicated groups)

To identify the specific role of FTH in HCC, we constructed the FTH overexpression HCC cells by transfecting HCCLM3 and MHCC97H cells with exogenous FTH overexpressed plasmid. The growth curves, either obtained by cell viability assay or real-time electrical impedance assay, showed that cells with FTH overexpression grew faster than the control cells, which was not restricted to a single cell lineage (Fig. 3A–C). Further, we tested the effect of FTH expression on cell invasion and migration, important elements for the malignancy of tumor. Results from transwell and wound—healing assays indicated that the expression of FTH increased the cell invasion and accelerated the healing of the wound (Fig. 3D–G). These results demonstrated that the overexpression of FTH could promote the proliferation, invasion and migration ability of HCC cells.

### FTH expression endows HCC cells specifically resistant to ferroptosis

**Fig. 4:**
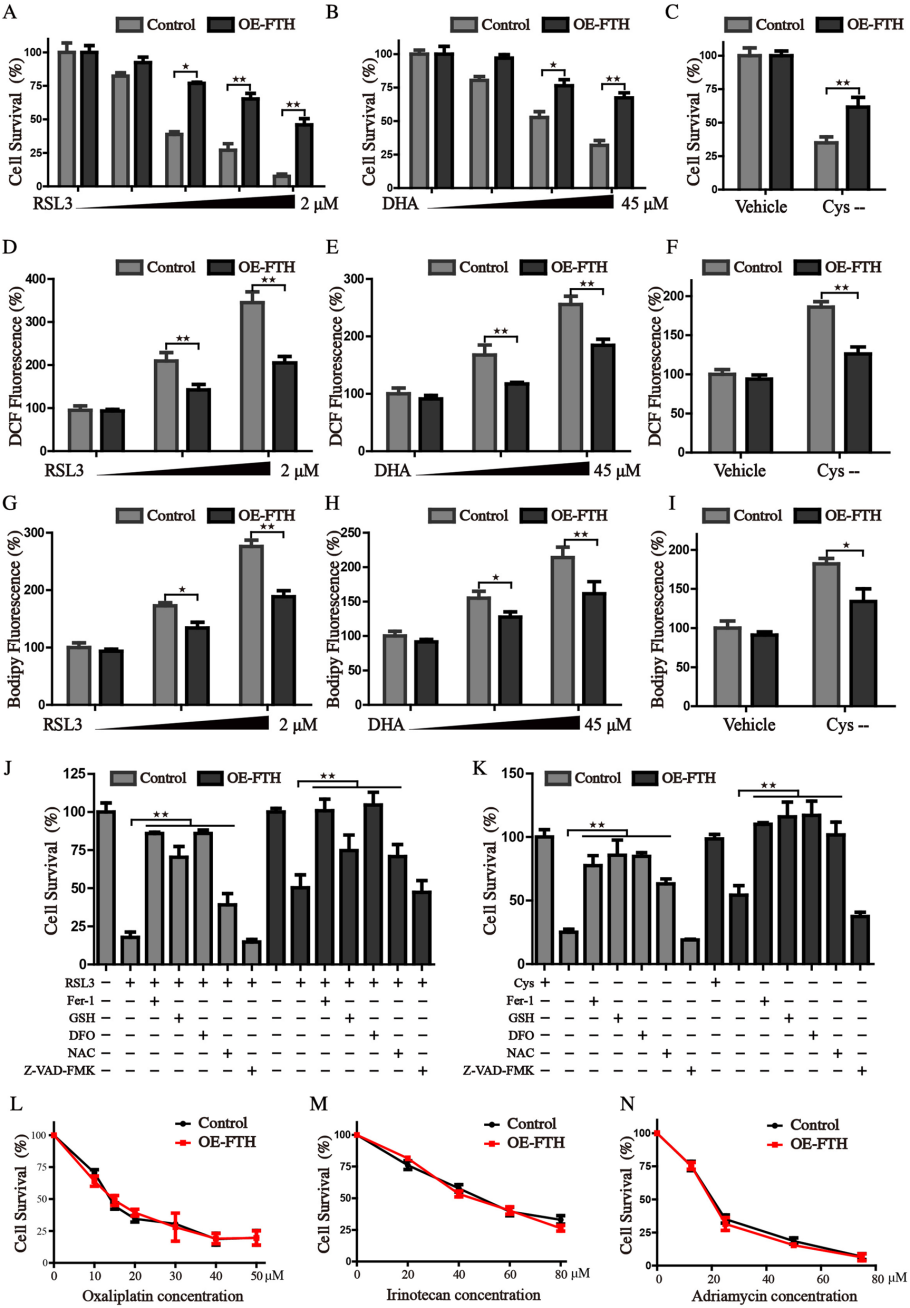
FTH inhibits the ferroptosis in HCC cells. **A**–**C** Control and FTH overexpressed cells were treated with RSL3 (0–2 μM), DHA (0–45 μM), or cystine-free medium, the cell survival rate was monitored by CCK8 assay. Cellular ROS (D-F) and lipid ROS production **G**–**I** were measured by the staining of DCF-DA and Bodipy after indicated treatment. **J**, **K** Control and FTH overexpression cells were treated RSL3 or cystine-free medium in the presence or absence of cell death inhibitors. The cell viability was detected by CCK8 assay. **L**–**N** FTH enforced expression and control cells were treated with Oxaliplatin (0–50 μm), Irinotecan (0–80 μm), or Adriamycin (0–72 μm) for 36 h, and the cell survival was measured using CCK8 assay. (Values are represented as mean ± SD. ^★^*P* < 0.05, ^★★^*P* < 0.01 versus indicated groups)

Since the aberrant accumulation of iron causes excess free radical generation through Fenton reaction, and subsequent results in significant alterations in cellular redox homeostasis. FTH has ferroxidase activity, and could catalytically oxidize Fe^2+^ to inactive Fe^3+^, which plays a vital role in maintaining iron homeostasis. Our recent papers have demonstrated that FTH plays a regulatory role in the initiation of ferroptosis [22, 23]. Ferroptosis is a newly identified programmed cell death, typically characterized by free iron overload and lethal phospholipid peroxide generation [11]. We thus hypothesized that high FTH expression might facilitate the resistance to ferroptosis via chelating free iron and give rise to the progression of HCC. Therefore, HCCLM3 cells with enforced expression of FTH were treated with several ferroptotic inducers. The results showed that FTH significantly ameliorated the ferroptotic cell death induced by RSL3, DHA and cystine depletion conditioned medium (Fig. 4A–C). Considering oxidative stress and lipid peroxidation are the driving factors for ferroptosis, we examined whether overexpression of FTH attenuated the oxidative stress. Indeed, the increased green fluorescence intensity of DCF-DA and BODIPY was detected after the treatment of ferroptotic inducers, but FTH expression significantly inhibited the formation of ROS and lipid peroxides (Fig. 4D–I). We treated control and FTH overexpression cells with RSL3 or cystine-free medium in the presence or absence of cell death inhibitors, including DFO (iron-chelating agent), ferrostatin-1 (ferroptosis inhibitor), Z-VAD-FMK (apoptosis inhibitor), GSH and NAC (antioxidant). And the results revealed that both ferroptosis inhibitors and FTH expression could significantly restore the process of ferroptosis (Fig. 4J, K). Furthermore, we found that FTH did not protect against cell death caused by cytotoxic compounds like oxaliplatin, irinotecan, and adriamycin, showing that FTH didn’t associate with canonical cell death mechanisms (Fig. 4L–N). Collectively, our results revealed that the protection against cell death conferred by FTH was specific to ferroptosis-inducing agents.

### FTH ameliorates iron stress and mitigates cellular oxidative damage

**Fig. 5:**
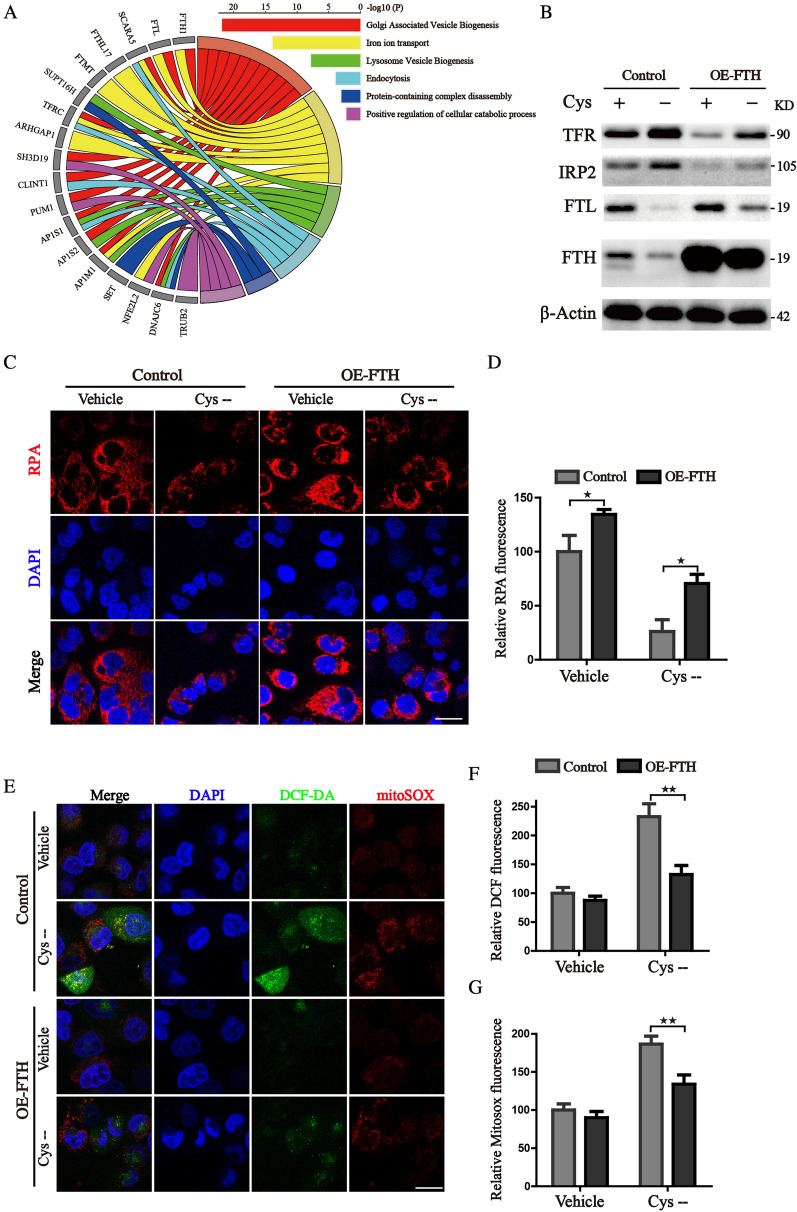
FTH protects the HCC cells from iron stress and oxidative damage. **A** The GO chord plot showed the top 18 significant FTH1-related genes and the 6 most enriched GO pathways (data from GeneMANIA database). **B** Control and FTH overexpression HCCLM3 cells were cultured with or without cystine-free medium and subjected to the western blotting assay for the detection of iron metabolism proteins. **C** Intracellular Fe^2+^ was examined by the staining of RPA probe and photographed by the confocal microscope (Scale bars 10 μm). **D** The quantitation of RPA fluorescence intensities was shown. **E** The staining of mitoSOX and DCF-DA was photographed by the confocal microscope (Scale bars 10 μm). **F**, **G** Corresponding quantitative histograms were presented. (Values are represented as mean ± SD. ^★^*P* < 0.05, ^★★^*P* < 0.01 versus indicated groups)

To better characterize the related events of FTH in the progression of hepatocellular carcinoma, we use the GENEMANIA database to analyze the proteins interaction network of FTH. As Fig. 5A shows, according to their score, the top 20 proteins were as follows: FTL, SCARA5, FTHL17, FTMT, SUPT16H, TFRC, ARHGAP1, SH3D19, CLINT1, PUM1, AP1S1, SIRT4, AP1S2, CSF3R, AP1M1, SET, NFE2L2, DNAJC6, TRUB2, and CEP55. PPI enrichment analysis was done to further identify the connected network components (Additional file 1: Fig. S1). We conducted pathway enrichment analysis of 20 genes related to FTH1 to perform predictive analysis through interactive visualization. The top 3 ranked pathways were Golgi-associated vesicle biogenesis, iron ion transport and lysosome vesicle biogenesis, demonstrating that FTH takes part in the maintaining of iron homeostasis and lysosomal dependent degradation. Then, cells were treated with cystine deprivation medium which acts as a ferroptosis inducer. The western blot analysis revealed that cystine deprivation mimics an iron-depleted cellular environment, promotes degradation of ferritin, and enhances iron uptake by upregulating TFR, thus increasing the free iron pool reflected by the fluorescence probe RPA and FerroOrange (Fig. 5B–D, Additional file 1: Fig. S2). Additionally, excessive accumulation of free iron significantly accelerated ferroptotic events including mitochondrial and cytoplasm ROS generation (Fig. 5E–G). However, enforced expression of FTH could attenuate the iron-depleted stress by storing iron in a soluble, non-toxic form (Fig. 5B–D, Additional file 1: Fig. S2). FTH reconstituted cells also exhibited reduced mitochondrial ROS lever and diminished peroxides accumulation when cultured in the cystine deprivation medium (Fig. 5E–G). Altogether, these results revealed that FTH promotes HCC cells' resistance to ferroptosis via attenuating free iron accumulation and peroxides generation.

### FTH rescues the mitochondrial homeostasis upon ferroptosis

**Fig. 6:**
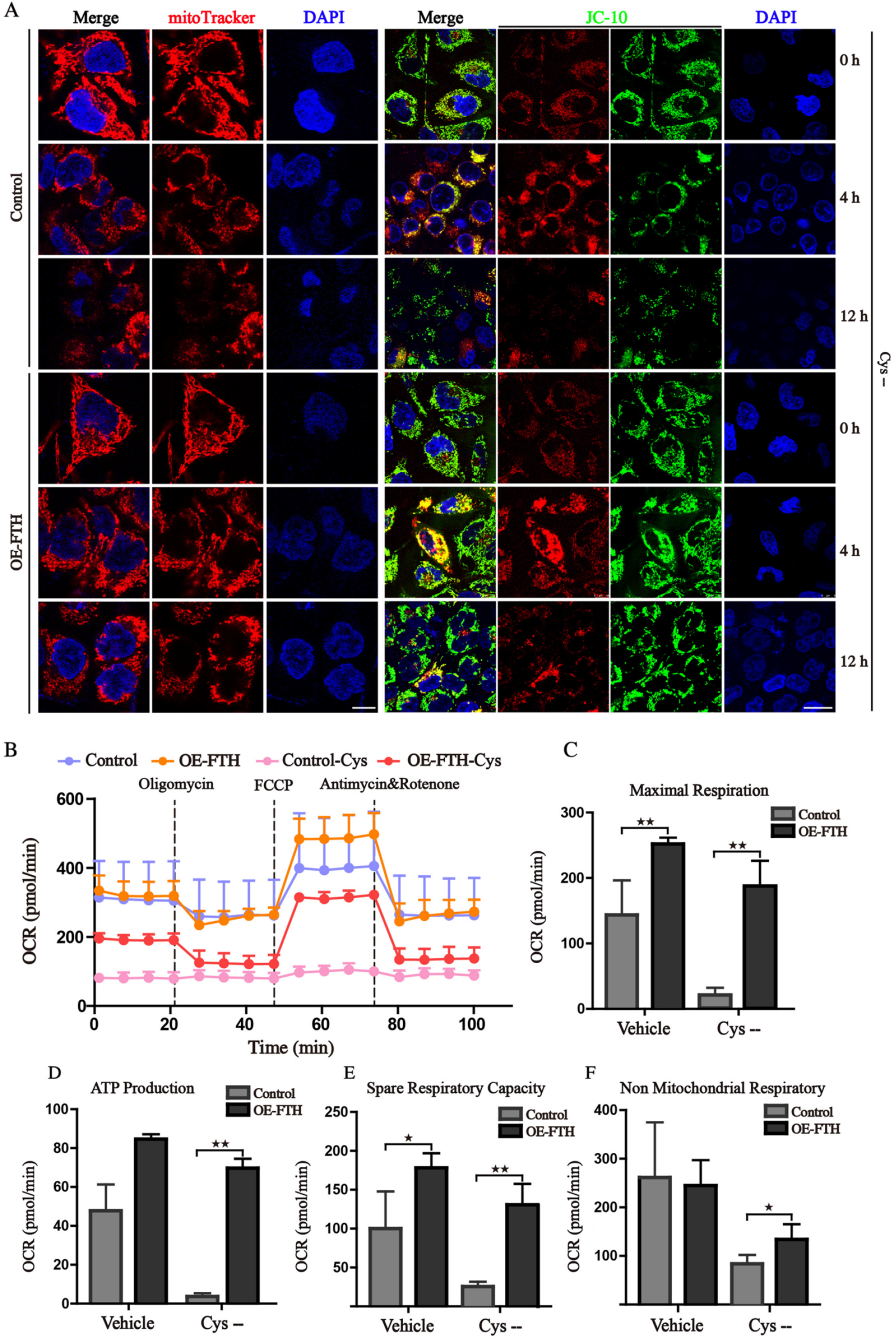
FTH rescues the impaired mitochondrial homeostasis upon ferroptosis. **A** Control or FTH overexpression HCCLM3 cells were treated with cystine-free medium, then stained by mitoTracker-Red and JC-10. The morphological changes of mitochondria and JC-10 fluorescence intensities were observed under confocal microscope (Scale bars 10 μm). **B** Control and FTH ectopic expression cells were treated with cystine-free medium for 8 h, the mitochondrial oxygen consumption rate (OCR) was monitored by Seahorse analyzer. **C**–**F** The OCR values of maximal respiration, ATP production, spare respiratory capacity, and non-mitochondrial respiratory were calculated. (Values are represented as mean ± SD. ^★^*P* < 0.05, ^★★^*P* < 0.01 versus indicated groups)

The highly soluble and reductive Fe^2+^ participates in various biological processes, such as iron-sulfur clusters assembly, mitochondrial respiration, and energy production [24]. Amplifying cancer cells harbor more labile Fe^2+^ mainly in mitochondria to meet the physiological need, which may produce hydroxyl radicals via the Fenton reaction [25]. Therefore, the high expression of FTH may act as a compensatory response to alleviate oxidative damage. We firstly examined the mitochondrial morphology and MMP, which have been demonstrated as a critical role in ferroptosis [22, 26]. Then, mitochondria visualized by mitoTracker red staining were characterized as fragmented and disorganized under the deprivation of cystine, while overexpression of FTH partly blocked the morphological changes mentioned above (Fig. 6A). In addition, we found that cystine deprivation temporarily induced MMP hyperpolarization, which was in line with previous research [27], whereas the MMP was significantly decreased after the treatment with cystine deprivation for 12 h. Enforced expression of FTH could stabilize the MMP and attenuate depolarization (Fig. 6A, Additional file 1: Fig. S3). As abnormality of mitochondrial morphology may interrupt the balance of mitochondrial oxidative phosphorylation, whose activity highly depends on the homeostasis of mitochondria. The maximal respiratory, spare respiratory capacity, ATP production and non-mitochondrial respiratory calculated by oxygen consumption rate (OCR) were all significantly inhibited with the deprivation of cystine (Fig. 6B–F). The declined respiratory may be the cause of MMP depolarization. Conversely, ectopic expression of FTH could attenuate the reduced mitochondrial respiratory and rescue the mitochondrial homeostasis. These results supported a mechanistic hypothesis that high expression of FTH acts as a protective role in HCC cells through alleviating oxidative damage and maintaining the homeostasis of mitochondria.

### FTH expression boost tumorigenic potential in vivo

**Fig. 7:**
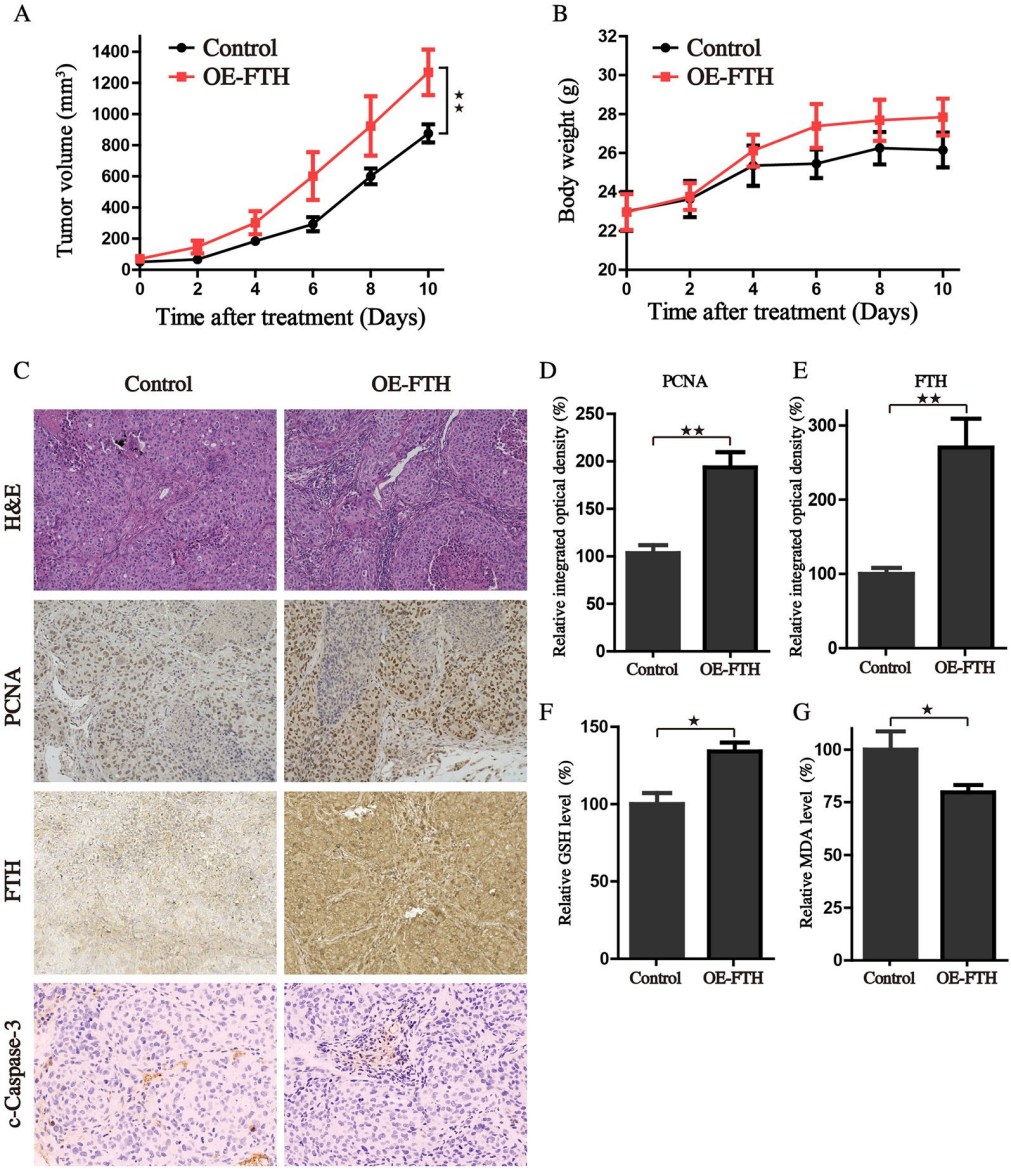
Over-expression of FTH promotes the growth of tumor xenograft in vivo. The tumor volume **A** and body weight **B** were detected every 2 days when tumors reached 80–100 mm^3^. **C** H&E and IHC staining for FTH, PCNA and c-Caspase-3 were applied in the indicated tumor tissue. **D** The IHC of PCNA and FTH was scored by relative integrated optical density (IOD) value and presented as histograms. (F-G) The relative GSH and MDA levels of indicated tumor tissues were measured by commercial kits, respectively. (Values are represented as mean ± SD. ^★^*P* < 0.05, ^★★^*P* < 0.01 versus indicated groups)

The subcutaneous tumor models bearing HCC cells were also employed to further investigate whether overexpression of FTH is essential for the enhanced tumor growth in vivo. Mirroring our in vitro data, the in vivo data further demonstrated that HCCLM3 cells with stable FTH overexpression significantly promote tumor growth in nude mice (Fig. 7A). The average body weight of mice did not change significantly in either group (Fig. 7B). These findings revealed that FTH plays a crucial role in promoting HCC cell growth in vivo. The tumors were detached from the mice in each group at the end of the experiment and experienced HE and IHC staining. Results showed that the tumor tissue with FTH enforced expression increased the PCNA staining (Fig. 7C–E). But the expression of cleaved caspase3 and FTL didn’t show obvious differences between the two groups (Fig. 7C, Additional file 1: Fig. S4). We next attempted to determine whether FTH boosts tumorigenic potential by lowering oxidative damage. The lysates from tumor tissues were analyzed, and the results revealed that FTH could increase the GSH level, with lesser lipid peroxides generation locally (Fig. 7F, G). Collectively, our findings showed that the FTH acts as an oncogene in the carcinogenesis and progression of HCC, and is hopeful to be a potential target for therapeutic intervention.

## Discussion

The last decades have witnessed a decrease in cancer mortality for the rapid developments of tumor diagnosis and comprehensive therapies. Different lethal regimens are designed to selectively target cancer cells via distinct programmed cell death processes with individual subroutines. Increasing evidence has demonstrated that inducing ferroptosis represents a promising therapeutic strategy to preferentially target iron-rich cancer cells such as HCC [28], NSCLC [29], PDAC [30] and breast cancer [31], and provides insights into reversing drug resistance in cancers. However, several endogenous antioxidant systems are compensatorily activated to render cells escaping from ferroptosis in a complex manner. For example, the GSH antioxidant system [32], CoQ10 dependent FSP1 antioxidants [33, 34], and FTH or ferroportin mediated iron metabolism [35, 36] are recognized as major protection systems to increase the cellular resistance to ferroptosis.

Iron is the most abundant metallic element and essential in living organisms on earth. Adult human male contains about 4 g of iron, which is incorporated into heme, Fe-S clusters and enzymes that are involved in the function of cellular respiration, electron transport, cell proliferation, and gene expression in cells. For the highly important role of iron during evolution, we do not harbor any active metabolic pathway to release iron from our bodies. However, overload of free iron is biochemically dangerous with the high capacity to promote the formation of ROS via the Fenton reaction, which results in peroxidation of membrane phospholipids and severe damage to the cellular biomolecules. To protect against vulnerability, cancer cells can exploit their defensive mechanisms. FTH is a major iron storing nanocage to store mineralized redox-inactive iron, and harbors ferroxidase activity to prevent the iron-mediated catalysis of ROS and tissue damage. In the current study, we discovered that FTH is significantly up-regulated in primary HCC tissues compared to normal samples, which is linked to high risk and poor prognosis in LIHC. Expression of FTH was significantly higher in more advanced cancer stages. Importantly, we also found that the level of serum ferritin is positively associated with the progression of hepatitis, cirrhosis liver and hepatocellular carcinoma. Our findings indicated that FTH expression is linked to high risk and poor prognosis in LIHC and might act as an oncogene in carcinogenesis and progression.

Besides lipid peroxidation, the accumulation of cellular free iron can significantly promote ferroptotic cell death. Based on recent reports, autophagic degradation of cellular FTH through ferritinophagy has been found to cause ferroptosis [37, 38]. Similarly, our previous work revealed that the dysfunction of iron homeostasis induced by dihydroartemisinin accelerated the onset of ferroptosis, which was consequently attenuated by FTH reconstitute expression [39]. TfR1 [40] (which imports iron from the extracellular environment into cells) and IRPs [41] (which regulate iron homeostasis through IRP-IRE mechanism), both contribute to iron starvation response and participate in the activation of ferroptosis. Our recent work revealed that knockdown of FXN activates iron starvation response and enhances erastin-induced ferroptosis through accelerating free iron accumulation. Blocking the iron starvation stress via pharmacological or genetic approaches restores the resistance to ferroptosis in FXN knockdown cells and xenograft [22]. Moreover, we found that overexpression of FTH could ameliorate the capacity of long-term cell viability of colony formation under erastin exposure, as well as prevent mitochondrial dysfunction. On the other hand, iron can trigger the production of highly toxic hydroxyl free radicals through Fenton reaction [8], and catalyze lipid peroxidation by converting arachidonic acid into leukotrienes [42]. Excess lipid peroxidation is particularly harmful to the dynamics of cell membranes, and precedes the complete bursting of the cell [43]. Therefore, the high expression of FTH may act as a compensatory response to alleviate oxidative damage.

To further evaluate whether FTH implicates the progression and aggressiveness of HCC, we enforced the expression of FTH in different HCC cell lines. Enhanced expression of FTH promoted the proliferation, invasion and migration ability of HCC cells, and boosted the tumorigenic potential of xenografts, demonstrating FTH regulates HCC tumor cells’ behavior both in vitro and in vivo. Notably, we found that FTH expression endows HCC cells specifical resistance to ferroptosis, whereas exhibits no protective effects on the cytotoxic compounds like oxaliplatin, irinotecan, and adriamycin, showing that FTH might represent a promising therapeutic target to preferentially induce HCC cells to ferroptosis.

As proliferating cells harbor catalytic Fe^2+^ mostly in the mitochondria, cellular iron levels and ROS homeostasis are tightly controlled to prevent toxic free iron accumulation and subsequent ROS production. Our recent work has found that the mitochondria-located protein CISD3 is vital for the regulation of iron metabolism [44]. Alteration of CISD3 expression impacts mitochondrial morphology, and regulates mitochondrial ROS production. Pamela et al. revealed that iron dyshomeostasis often accompanies mitochondrial dysfunction. Knockdown of IRP1 leads to increased FTH expression and a lower iron labile pool, which gives rise to the resistance to cystine oxidation [45]. Therefore, we addressed the question that whether the expression of FTH increased the aggressive features of HCC cell lines by ameliorating the mitochondrial dysfunction. Our results elucidated that FTH could attenuate the iron-depleted stress and rescue the mitochondrial function, which was characterized as follows: (i) FTH mitigated the changes in mitochondrial morphology monitored by mitoTracker probe; (ii) FTH could stabilize the MMP and attenuate the depolarization under the challenge of cystine deprived cultivation; (iii) ectopic expression of FTH could attenuate the reduced mitochondrial respiratory, reduce the mitochondrial ROS generation and rescue the mitochondrial homeostasis. These results supported that high expression of FTH acts as a protective role in HCC cells through alleviating the mitochondrial dysfunction and ameliorating ferroptosis.

Altogether, our study provides evidence that FTH participates in the carcinogenesis and progression of hepatocellular carcinoma through modulating iron metabolism and maintaining mitochondrial homeostasis. Overexpression of FTH has been proposed to increase the malignancies of HCC cells, which could represent a novel prognostic biomarker and a potential therapeutic target. We also provide a framework for further understanding and targeting ferroptosis in cancer therapy.

## Supplementary Information

**Additional file 1: Fig. S1.** PPI network construction of FTH1 correlating genes.

**Additional file 2: Fig. S2.** Mitochondrial Fe^2+^ was examined by the staining of FerroOrange probe and photographed by the confocal microscope (Scale bars 10 μm).

**Additional file 3: Fig. S3.** Flow cytometric analysis of mitochondrial membrane potential through TMRM staining.

**Additional file 4: Fig. S4.** The western blotting assay detected the expression level of FTL and HO-1.

## Data Availability

All data generated during this study are included either in this article or in the supplementary information files.

## Abbreviations

CHOL: Cholangio carcinoma
HNSC: Neck squamous cell carcinoma
KIRC: Kidney renal clear cell carcinoma
KIRP: Kidney renal papillary cell carcinoma
LIHC: Liver hepatocellular carcinoma
THCA: Thyroid carcinoma
LGG: Brain Lower Grade Glioma
LAML: Acute Myeloid Leukemia
KICH: Kidney chromophobe
THYM: Thymoma
OS: Overall survival
HCC: Hepatocellular Carcinoma
FTH: Ferritin heavy chain
ROS: Reactive oxygen species
GPX4: Glutathione peroxidase 4
NRF2: Nuclear factor erythroid 2-related factor 2
TFR: Transferrin receptor protein
TFR: Transferrin receptor
FTL: Ferritin light chain
NCOA4: Nuclear receptor coactivator 4
GSH: Glutathione
