# Supplementary figures and images for: FTH promotes the proliferation and renders the HCC cells specifically resist to ferroptosis by maintaining iron homeostasis
Source: Cancer Cell Int. 2021 Dec 29;21:709. doi: 10.1186/s12935-021-02420-x (PMC8717654; doi:10.1186/s12935-021-02420-x)

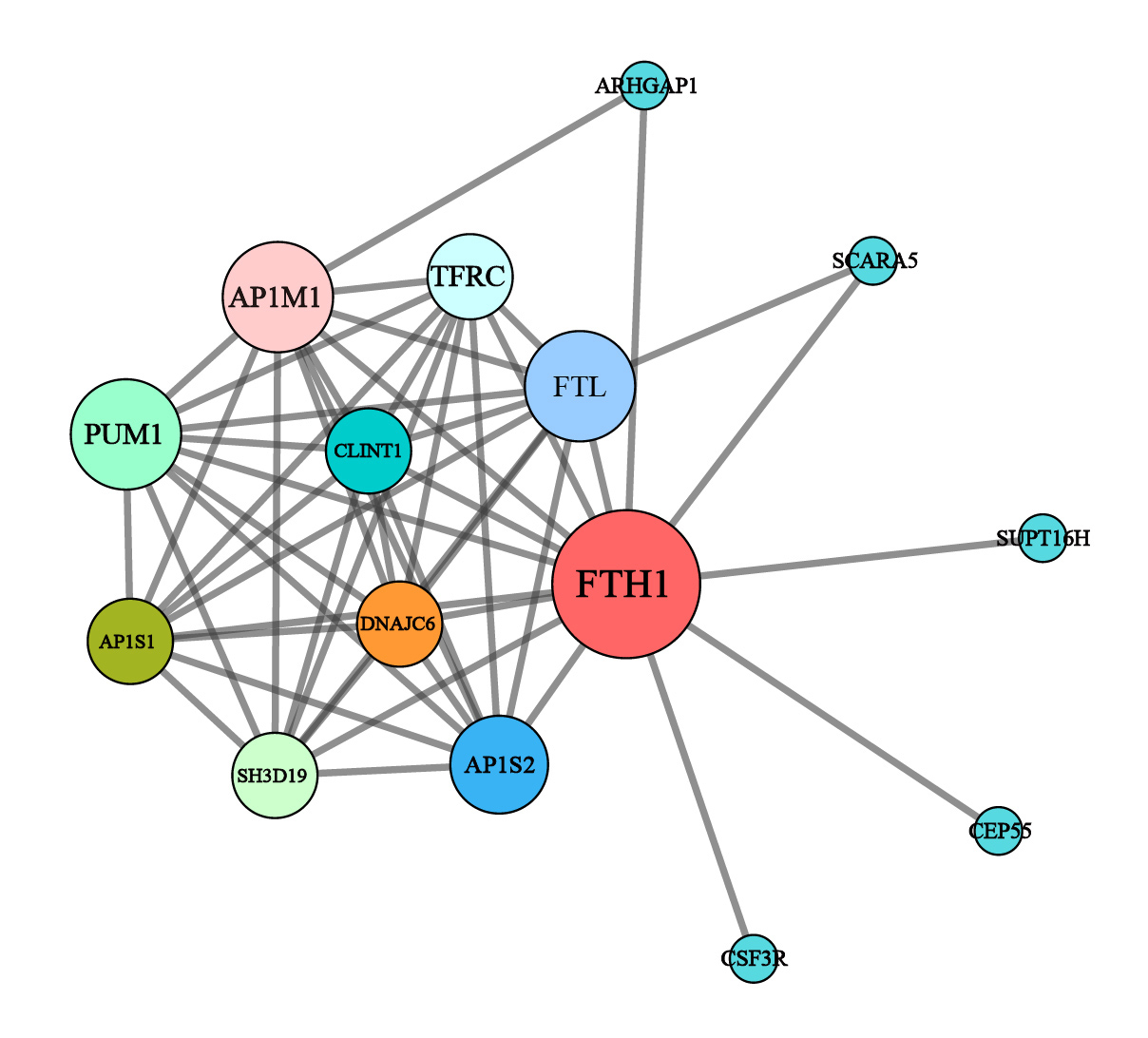

Supplement: Supplementary file 1 — Additional file 1: Fig. S1. PPI network construction of FTH1 correlating genes. [file 12935_2021_2420_MOESM1_ESM.jpg]

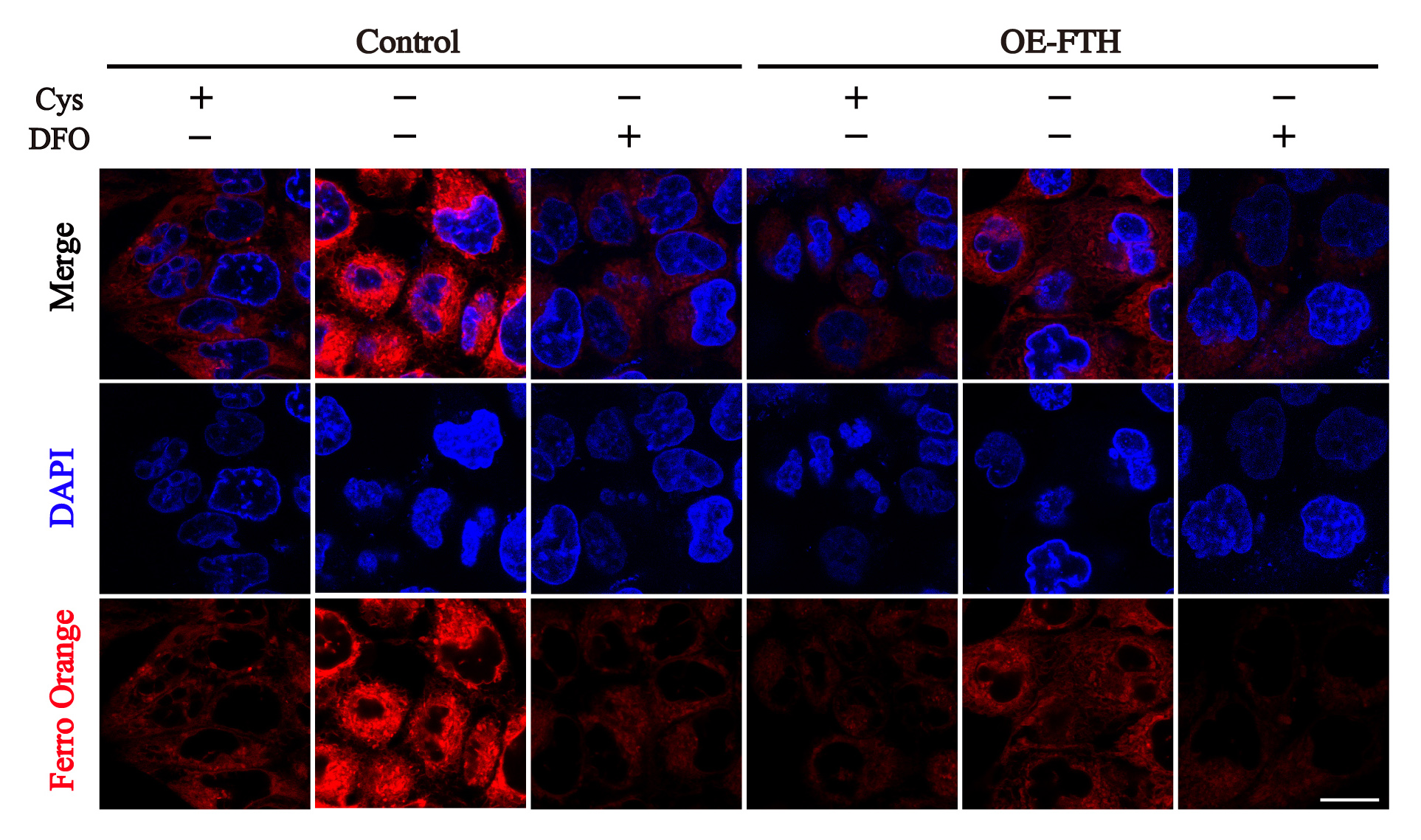

Supplement: Supplementary file 2 — Additional file 2: Fig. S2. Mitochondrial Fe2+ was examined by the staining of FerroOrange probe and photographed by the confocal microscope (Scale bars 10 μm). [file 12935_2021_2420_MOESM2_ESM.jpg]

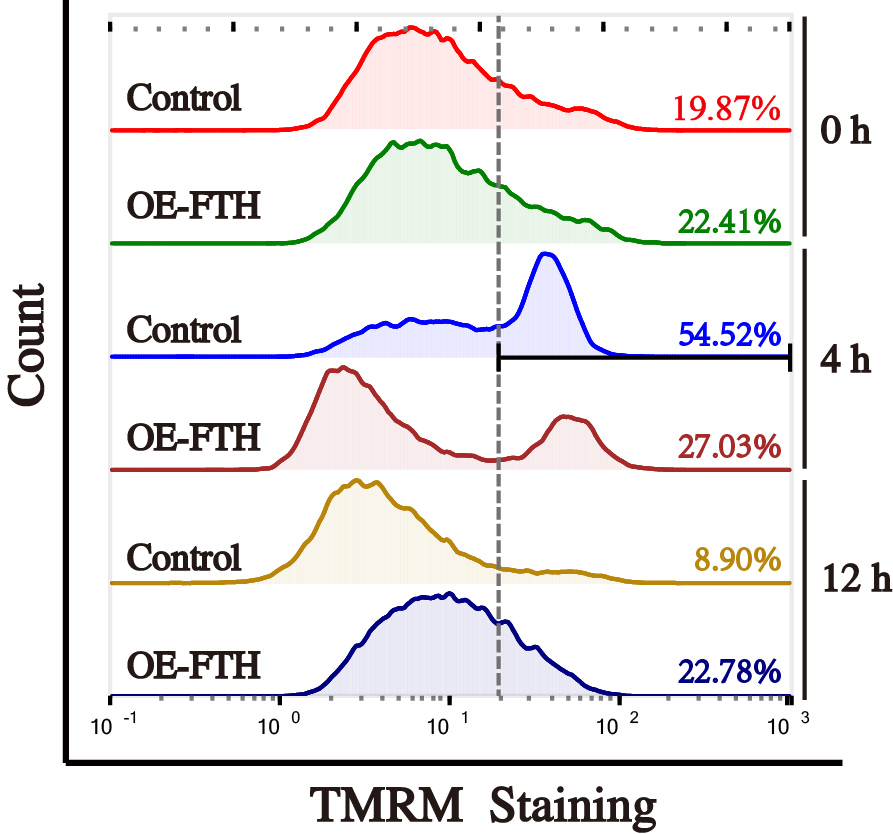

Supplement: Supplementary file 3 — Additional file 3: Fig. S3. Flow cytometric analysis of mitochondrial membrane potential through TMRM staining. [file 12935_2021_2420_MOESM3_ESM.jpg]

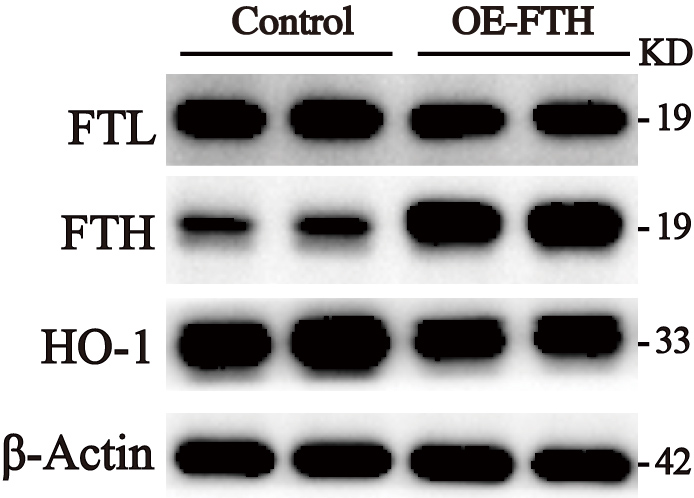

Supplement: Supplementary file 4 — Additional file 4: Fig. S4. The western blotting assay detected the expression level of FTL and HO-1. [file 12935_2021_2420_MOESM4_ESM.jpg]
